# Best practices for secondhand smoke and secondhand aerosol protection and evidence supporting the expansion of smoke- and aerosol-free environments: Recommendations from the 2nd Joint Action on Tobacco Control

**DOI:** 10.18332/tpc/193147

**Published:** 2024-10-21

**Authors:** Irene Possenti, Silvano Gallus, Alessandra Lugo, Anna Mar López, Giulia Carreras, Raquel Fernández-Megina, Adrián González-Marrón, Giuseppe Gorini, Helena Koprivnikar, Efstathios Papachristou, Angeliki Lambrou, Sotiria Schoretsaniti, Melinda Pénzes, Dolors Carnicer-Pont, Esteve Fernandez, on behalf of JATC-2 WP8 Partners

**Affiliations:** 1 Department of Medical Epidemiology, Istituto di Ricerche Farmacologiche Mario Negri, Milan, Italy; 2 Tobacco Control Unit, WHO Collaborating Centre for Tobacco Control, Catalan Institute of Oncology (ICO), L'Hospitalet de Llobregat, Barcelona, Spain; 3 Tobacco Control Research Group, Institut d’Investigacio Biomedica de Bellvitge (IDIBELL), l’Hospitalet de Llobregat, Barcelona, Spain; 4 Centre for Biomedical Research in Respiratory Diseases (CIBERES), Institute of Health Carlos III, Madrid, Spain; 5 Oncologic Network, Prevention and Research Institute (ISPRO), Florence, Italy; 6 Association NoFumadores.org, Madrid, Spain; 7 Group of Evaluation of Health Determinants and Health Policies, Faculty of Medicine and Health Science, International University of Catalonia, Barcelona, Spain; 8 National Institute of Public Health, Ljubljana, Slovenia; 9 Directorate of Epidemiology and Prevention of Non- Communicable Diseases and Injuries, National Public Health Organization (NPHO), Athens, Greece; 10 Health Services Management Training Centre, Semmelweis University, Budapest, Hungary; 11 Department of Clinical Sciences, School of Medicine and Health Sciences, University of Barcelona, Barcelona, Spain

**Keywords:** smoke-free regulation, secondhand smoke, secondhand aerosol, smoke-free environment, aerosol-free environment

Exposure to secondhand tobacco smoke (SHS) is a global health threat that causes diseases and kills more than 1.2 million people each year, including 65000 children^1-4^. The World Health Organization (WHO) Framework Convention on Tobacco Control (FCTC) urges countries to establish comprehensive smoke-free environments^5^. Although European Union (EU) member states have implemented smoke-free laws, challenges persist with designated smoking rooms (e.g. in hospital venues and in airports) and neglect of emerging products, such as electronic cigarettes (e-cigarette) and heated tobacco products (HTP)^6,7^. This editorial provides concise recommendations on smoke- and aerosol-free environments (SAFE) in the EU, focusing on evidence-based strategies for SAFE. Promoting the expansion of SAFE throughout EU countries was a key objective of the JATC-2, a project co-funded by the European Commission. To address this objective, WP8 focused on the current framework and potential expansion of SAFE in Europe. As part of this effort, a consultation was conducted in 2022, engaging 110 experts from 27 EU member states, along with Norway, Serbia, and the United Kingdom. The sources to identify experts were the JATC-2 contact list of all authorities and stakeholders working with tobacco regulation (policymakers and regulators, researchers and tobacco inspectors) for countries of the EU; the Catalan Institute of Oncology/WHO Collaborating Center for Tobacco Control list of contacts, including speakers and attendees to five editions of ICO-WHO Symposia on tobacco control; and lists of contacts from Smoke-Free Partnership and the European Network for Smoking and Tobacco Prevention. The responses from these experts provided valuable information on the barriers, opportunities, and best practices associated with SAFE policies across different countries. This collective knowledge, combined with findings from a systematic literature review (covering articles published between January 2010 and August 2022) and dedicated discussions (including a symposium satellite of the 9th European Conference on Tobacco or Health), forms the basis for recommendations on effective strategies and interventions to safeguard individuals from SHS and secondhand aerosol (SHA). The expert consultations are summarized in the following recommendations.

## Complete ban – without exemptions – and enforcement for indoor and outdoor environments

### Indoor and outdoor workplaces (public and private)

Enforcing strong tobacco and nicotine use bans (hereafter referred to as smoking bans) in public and private workplaces, both indoors and outdoors, is essential to ensure the health and well-being of workers. Despite widespread support, compliance remains a challenge, particularly outdoors, and enforcement varies across EU countries^8-10^.

The impact of smoking on lost productivity, increased sick days and reduced concentration^11,12^ underscores the need for a smoke-free environment that benefits both employees and customers, smokers and non-smokers. Additionally, fire hazards posed by smoking materials and improperly discarded cigarette butts should also be considered. Best practices from Denmark, such as the ‘Smoke-free work hours’ policy, demonstrate effectiveness, emphasizing the need for consistent and stringent enforcement in most EU countries^13^. Complete smoking bans not only protect employees from SHS and SHA, but also promote a healthier work environment, encouraging smokers to quit or reduce their cigarette consumption^6,14^.

### Indoor and outdoor hospitality venues (public and private)

In the EU, indoor smoking bans in hospitality venues show good compliance, but challenges persist outdoors, where many countries have partial or no legislation^15^. Exclusive indoor bans lead smokers outdoors, increasing nicotine exposure in bar and restaurant terraces^16,17^.

Bans in public places provide positive role models, particularly for young people, sending a clear message against the social acceptability of tobacco and nicotine use. This may discourage smoking initiation and supports cessation efforts^18-20^. Complete bans are essential, acknowledging no safe exposure level to SHS/SHA^7,21^.

### Indoor and outdoor public transport

Implementing smoking bans in public transport (train, bus, airports), including outdoor areas, improves safety and accessibility for passengers. Notable examples from Hungary, the Netherlands, and Estonia demonstrate success, with numerous train stations and various modes of transport becoming smoke-free^22^. Outdoor smoke-free policies not only contribute to passenger well-being but also help to reduce PM2.5 levels and litter caused by discarded cigarette butts, which is a major global concern^23,24^. Governments and communities should recognize these benefits, emphasizing the need for complete smoking bans in outdoor places related to public transport and reinforcing indoor bans.

### Indoor and outdoor settings frequented by minors, swimming pools, and sport settings

EU countries have implemented smoking bans in public spaces frequented by children, but legislation remains partial or absent in about half of the EU countries^15^. Recent studies show concerning findings, including the presence of airborne nicotine and cigarette butts in EU playgrounds^25,26^. Despite challenges, there is strong support for implementing smoke-free policies in outdoor settings for children^27^. The primary motivation is to protect children from the harmful effects of SHS/SHA, as they are more susceptible to respiratory issues and other health problems^3^. In addition, enforcing smoking bans in these areas significantly deters youth from starting smoking^7,18^. The Luxembourg model, featuring smoke-free children’s playgrounds, is a notable best practice identified in the consultation within WP8^28^. Numerous outdoor sport clubs in the Netherlands, Spain, and other European countries that have voluntarily adopted smoke-free policies are positive examples of creating healthier environments^29^. Similarly, introducing and enforcing smoke-free policies in swimming pools across Europe would further contribute to protect the health and well-being of minors and adults in recreational and sport settings.

### Indoor and outdoor healthcare facilities

While most EU countries have implemented complete indoor smoking bans in healthcare facilities, the scenario differs outdoors, with generally partial or no bans across almost all EU member states^15^. Enforcing smoking bans in healthcare facilities (indoor and outdoor) is crucial for maintaining clean and healthy environments, particularly for vulnerable individuals^23^. Healthcare professionals, as role models, have an important influence on the promotion of healthy habits. The Ireland strict ban on smoking in ‘The Health Service Executive facilities’ sets a notable example identified by WP8, serving as a model for future developments and emphasizing strong support for smoking bans in healthcare facilities^30^.

### Private vehicles

Smoking in cars or driving motorcycles poses a distraction for drivers, compromising road safety^31,32^. Legislation for smoke-free private cars aims at reducing the risk and ensures the well-being and safety of both smokers and passengers. There is strong support for smoke-free policies in cars, especially in the presence of minors, given the well-recognized danger of SHS/SHA to children’s health^27^. Several EU countries have enacted laws prohibiting smoking in cars with minors^33-37^. Smoke can reach high levels even with the windows open, posing important health risks^38^. Moreover, the environmental threat of fires is increased as smoking drivers may discard cigarette butts along the route^39^.

### Selected outdoor settings, including parks, forests, and beaches

Despite the potentially high levels of SHS/SHA exposure in these frequented outdoor spaces, only a few EU countries have comprehensive bans in these areas. Successful bans in beaches such as Bibione (Italy)^26,40^, and Barcelona, Catalonia (Spain)^41^, demonstrate their positive impact. These bans encourage healthier behaviors and preserve natural spaces from discarded cigarette butts, which pose toxic risks to soil and water. In addition, they reduce the likelihood of accidental fires^21,42^. Effective implementation requires raising awareness through targeted campaigns and communication strategies, as well as collaboration among local authorities, community organizations, stakeholders, and environmental groups. Implementing smoking bans in selected outdoor areas, such as parks, forests, and beaches is crucial for creating healthier environments and safeguarding public health.

### Public housing and multi-unit dwellings

Public housing and multi-unit dwellings are prime candidates for implementing smoking bans due to the ease with which SHS can spread between apartments, corridors, and community rooms. A study in Denmark revealed that 22% of people living in multi-unit dwellings were exposed to neighbors’ smoke, with 58% of exposed people in favor of smoke-free multi-unit dwellings^43^. A systematic review of 35 studies in the USA suggests strong support for smoke-free multi-unit dwelling policies among most residents^44^.

## Voluntary smoking ban for homes: Avoid exposure to vulnerable populations, including minors

Promoting voluntary smoke-free homes through information campaigns protects vulnerable populations, especially minors, from SHS. Government smoke-free campaigns have significantly increased smoke-free homes, reducing SHS exposure and diminishing the social acceptability of smoking^24,45,46^. The implementation of indoor smoking bans improves air quality, reducing PM10 and PM2.5 levels^47,48^. In addition to the health benefits, avoiding smoking in the home minimizes fire hazards and creates a safer environment. A voluntary smoking ban at home sets a positive example for children, fostering healthy behaviors and a smoke-free lifestyle^49^. The successful ‘Smoke-free Homes – Take 7 steps out’ campaign in the United Kingdom serves as inspiration^50^, emphasizing the importance of motivating individuals and families to actively choose smoke-free private spaces for a healthier future.

## Equalizing legislation for electronic cigarettes and heated tobacco products to that of conventional tobacco products

E-cigarettes and HTPs have grown in popularity^51,52^. However, the scientific community, including the WHO, poses health concerns regarding these products, given the development of respiratory disorders associated with e-cigarette use and potential toxic emissions from HTPs. Current evidence does not support reduced health risks from HTPs compared with conventional cigarettes^53^. Although a Cochrane review suggests that e-cigarettes gave higher quit rates than nicotine replacement therapy, success remains low, with over 80% of users continuing to use e-cigarettes after quitting smoking^54^. Moreover, a recent Italian cohort study confirmed that the use of e-cigarettes and HTPs may be associated with smoking initiation among never smokers, especially young people, and relapse among former smokers^55^, in addition to accumulated evidence in the last decade^56-58^.

The dynamic nature of these products challenges regulation, allowing rapid market expansion with unknown long-term effects. This raises concerns for public health and consumer protection, undermining efforts to de-normalize smoking.

European regulation is inconsistent, with only a few countries that have equalized smoking legislation for e-cigarettes and HTPs. This has led smokers to use e-cigarettes or HTPs indoors, resulting in a majority of dual users and exposing a substantial portion of the population to SHA, exceeding the levels found for SHS.

## Barriers and opportunities for the expansion of SAFE

The barriers to the expansion of SAFE that were identified included: the pervasive influence of the tobacco industry, driven by lobbying and funding activities; government reluctance, in the form of inadequate outdoor legislation, lax vending regulations, and a false sense that problems related to smoking and tobacco control have been solved; resistance to SAFE policies of specific settings, including hospitality, tourism, small businesses, and private homes; misinformation, resulting from a lack of accurate data on tobacco nicotine-containing products and concerns over insufficient evidence of harm and about stigmatizing smokers.

However, numerous opportunities for the expansion of SAFE also arise such as: extending SAFE policies to specific outdoor venues; enhancing awareness; supporting educational initiatives; promoting transparency; imposing significant fines; and focusing on clear strategies that can effectively advance SAFE policies. Barriers and opportunities for the expansion of SAFE policies are presented in one of the research articles in this special issue.

## Conclusion

Promoting SAFE in the EU is vital for protecting public health and reducing the harm caused by tobacco and nicotine products. Comprehensive smoke-free regulations covering indoor and outdoor settings, including private vehicles along with advocating for voluntary smoke-free homes, are essential steps. Equalizing legislation for emerging tobacco products (HTPs and all e-cigarettes, including non-nicotine variants) with conventional cigarettes is crucial for public health protection. Finally, enforcement of smoke-free laws, civil society engagement, and robust monitoring promise to increase the impact of SAFE legislation.

## Data Availability

The data supporting this research are available from the authors on reasonable request.
